# Short‐ and Midterm Analgesic Outcomes of Ketamine Versus Lidocaine Infusion in Chronic Peripheral Neuropathic Pain: A Retrospective Study

**DOI:** 10.1155/prm/7897619

**Published:** 2026-09-01

**Authors:** Mevlüt Gökhan Sucu, Tuğçe Yavuz Mollavelioğlu, Ayşegül Akyüz Yildirim, Nalan Çelebi

**Affiliations:** ^1^ Department of Anesthesiology, Pain Medicine, Hacettepe University Medical School, Ankara, Türkiye, hacettepe.edu.tr; ^2^ Department of Pain Medicine, Mersin City Training and Research Hospital, University of Health Sciences, Mersin, Türkiye, akdeniz.edu.tr; ^3^ Department of Pain Medicine, Gaziosmanpaşa Training and Research Hospital, Istanbul, Türkiye

**Keywords:** chronic pain, infusions, intravenous, ketamine, lidocaine, neuropathic pain, pain measurement

## Abstract

**Background:**

Intravenous ketamine and lidocaine infusions are increasingly used for chronic peripheral neuropathic pain, particularly in refractory cases. However, comparative data on their short‐ and midterm analgesic outcomes in routine clinical practice are limited.

**Methods:**

This retrospective study included patients with chronic peripheral neuropathic pain who received intravenous ketamine or lidocaine infusion between January and May 2021. Ketamine was administered at 1 mg/kg/h and lidocaine at 3 mg/kg/h for five consecutive days. Pain intensity and neuropathic pain features were assessed using the Numeric Rating Scale (NRS) and the Douleur Neuropathique 4 (DN4) questionnaire at baseline and at 2 weeks, 2 months, and 6 months after treatment. Clinically meaningful pain relief was defined as a ≥ 50% reduction in pain scores.

**Results:**

A total of 102 patients were analyzed (ketamine group, *n* = 44; lidocaine group, *n* = 58). Both groups showed significant reductions in NRS and DN4 scores at all follow‐up points compared with baseline (*p* < 0.01). Clinically meaningful pain relief was more frequent in the ketamine group at 2 weeks (*p* < 0.01), whereas no significant differences were observed at 2 and 6 months (*p* > 0.05). Adverse effects occurred more often with ketamine, while lidocaine was associated with fewer side effects (*p* < 0.05).

**Conclusion:**

Ketamine provided a greater early analgesic response at 2 weeks, whereas both treatments demonstrated comparable midterm efficacy at 2 and 6 months.

**Trial Registration:** ClinicalTrials.gov identifier: NCT06297915

## 1. Introduction

Neuropathic pain (NP) is defined by the International Association for the Study of Pain (IASP) as pain arising as a direct consequence of a lesion or disease affecting the somatosensory nervous system, involving either central or peripheral components [1]. The prevalence of NP in the general population has been reported to be approximately 7%–10%, representing a substantial burden on both patients and healthcare systems [2]. According to the updated IASP classification, trigeminal neuralgia, peripheral nerve injury, painful polyneuropathy, postherpetic neuralgia, and painful radiculopathy are among the primary causes of peripheral NP [3]. Clinically, peripheral NP is characterized by spontaneous and evoked symptoms, including allodynia, hyperalgesia, paresthesia, tingling sensations, and numbness [4].

Both pharmacological and nonpharmacological approaches are utilized in the management of NP. First‐line pharmacological treatments include tricyclic antidepressants (TCAs), serotonin–noradrenaline reuptake inhibitors (SNRIs), and gabapentinoids. Second‐line options comprise weak opioids, topical lidocaine, and capsaicin, whereas strong opioids are generally reserved for third‐line therapy [5]. Nonpharmacological strategies, such as interventional pain management techniques, physical therapy modalities, and psychological interventions, may also be incorporated into a multimodal treatment plan [5]. Despite these therapeutic options, NP remains challenging to manage, particularly in patients with severe or refractory symptoms. Previous studies have shown that adequate pain relief cannot be achieved in more than 50% of patients receiving conventional treatments [6]. Moreover, treatment efficacy is frequently limited by adverse effects, resulting in poor tolerability and reduced adherence to long‐term pharmacotherapy [5]. These limitations have driven the search for alternative treatment strategies targeting mechanisms of central sensitization.

In recent years, systemic infusion therapies using ketamine and lidocaine have gained increasing attention in the management of NP. Ketamine, a noncompetitive N‐methyl‐D‐aspartate (NMDA) receptor antagonist, is administered at subanesthetic doses for both acute and chronic pain conditions. Beyond NMDA receptor antagonism, ketamine has been suggested to modulate descending inhibitory pathways and exert anti‐inflammatory effects within the central nervous system; however, these mechanisms have largely been demonstrated in preclinical studies and require further confirmation in humans [7, 8]. Lidocaine, a nonselective sodium channel blocker, produces systemic analgesia by suppressing ectopic neuronal firing and may attenuate central sensitization through modulation of glial activity in the central nervous system, although this effect has been demonstrated primarily in preclinical studies and its clinical relevance in humans remains to be established. The analgesic effect of intravenous lidocaine has generally been reported as short‐lived; the onset of action is estimated at 30–60 min, with effects typically lasting 2–6 h following the end of infusion [9, 10].

The aim of this study was to evaluate and compare the short‐term (2 weeks) and midterm (2 and 6 months) analgesic efficacy and safety of intravenous ketamine and lidocaine infusions in patients with chronic peripheral NP. The primary objective was to evaluate the change in Numeric Rating Scale (NRS) pain scores at 2‐week follow‐up. Secondary objectives were to evaluate changes in Douleur Neuropathique 4 (DN4) scores across all follow‐up time points (2 weeks, 2 months, and 6 months), changes in NRS scores at 2‐month and 6‐month follow‐up, and the incidence of adverse effects in both treatment groups.

## 2. Materials and Methods

### 2.1. Study Design and Patient Selection

This retrospective study was approved by the local ethics committee of Hacettepe University Medical School (May 10, 2022; No. 2022/08‐45), and written informed consent was obtained from all patients prior to treatment. Medical records from the electronic hospital database and patient files were reviewed for individuals admitted to the algology clinic with symptoms of NP between January 2021 and May 2021.

Patients aged 18–65 years with a diagnosis of peripheral neuropathic pain with symptom duration of more than 3 months, including radicular neuropathy, diabetic neuropathy, complex regional pain syndrome type 2 (CRPS type 2), traumatic peripheral nerve injury, trigeminal neuralgia, postherpetic neuralgia, or non–cancer‐related peripheral neuropathic pain, and a DN4 score ≥ 4 at presentation, were included.

Exclusion criteria comprised central NP patterns or previous central nervous system disease, renal, hepatic, cardiovascular, or psychiatric comorbidities, incomplete treatment due to adverse effects or other reasons, and loss to follow‐up.

### 2.2. Clinical Assessment

Demographic characteristics (age, sex), clinical data (comorbidities, concomitant medications), and anthropometric parameters (body weight and height) recorded by the algology physician before treatment were collected. Ongoing pain treatments were reviewed, and infusion therapy was initiated without discontinuation of baseline medications. Pain intensity was assessed using the NRS, in which patients rated their average pain intensity over the preceding 24 h on a scale from 0 (*no pain*) to 10 (*worst imaginable pain*). Assessments were performed at baseline (prior to the first infusion) and at 2 weeks, 2 months, and 6 months following the completion of the infusion protocol. The DN4 questionnaire was administered at the same time points to monitor changes in NP characteristics. It should be noted that the DN4 questionnaire was used as a screening tool to support the clinical diagnosis of NP, with a score ≥ 4 indicating probable NP, demonstrating a sensitivity of 82.9% and specificity of 89.9% in the Turkish validation study [11]. Definitive diagnosis was established by the treating physician based on clinical history, neurological examination, and the underlying etiology of each patient.

### 2.3. Infusion Protocol and Safety Monitoring

Patients received intravenous ketamine or lidocaine infusion after being informed about the procedure and providing consent. All infusions were administered under the supervision of a physician and nurse. Both treatment groups received infusion therapy for five consecutive days. Lidocaine was administered at a dose of 3 mg/kg/h diluted in 150 mL of isotonic saline, while ketamine was administered at a dose of 1 mg/kg/h diluted in 150 mL of isotonic saline. Each infusion session lasted 1 h. The ketamine dose was selected based on published subanesthetic dosing recommendations for chronic NP [7], while the lidocaine dose of 3 mg/kg was chosen in accordance with previously reported effective doses in NP studies and to ensure an acceptable safety profile in an outpatient clinical setting [10]. Vital signs and adverse effects were monitored throughout the infusion period. Infusion was discontinued in cases of life‐threatening vital sign changes, hypersensitivity reactions, or patient request. Adverse effects were assessed clinically by the supervising physician during and after each infusion session. Mild adverse effects were defined as transient, self‐limiting symptoms requiring no intervention, while moderate adverse effects were defined as symptoms requiring symptomatic treatment but not discontinuation of the infusion. Recorded adverse effects included psychotomimetic symptoms (dissociation, hallucination), cardiovascular changes (hypertension, tachycardia), neurological symptoms (dizziness, headache), and gastrointestinal symptoms (nausea, vomiting). Patients were observed for 1 h following each infusion, and delayed adverse events were documented.

### 2.4. Follow‐Up

After completion of infusion therapy, patients were evaluated at outpatient clinic visits at 2 weeks, 2 months, and 6 months. DN4 and NRS scores, along with clinical follow‐up data, were recorded at each visit.

### 2.5. Statistical Analysis

Statistical analyses were performed using SPSS Version 26.0 (IBM Corp., USA). Categorical variables were expressed as frequencies and percentages. Normality was assessed using both the Kolmogorov–Smirnov and Shapiro–Wilk tests, performed separately within each treatment group. DN4 and NRS scores at all time points showed non‐normal distributions in both groups (*p* < 0.05), supporting the use of nonparametric statistical methods. Age was normally distributed in both groups (*p* > 0.05), whereas BMI and pain duration deviated from normality in at least one group. Nonnormally distributed continuous variables were presented as median and interquartile range (IQR, 25th–75th percentiles).

Between‐group comparisons for nonnormally distributed variables were conducted using the Mann–Whitney *U* test. Repeated measures of DN4 and NRS scores were analyzed using the Friedman test. Clinically meaningful pain relief was defined as a ≥ 50% reduction in DN4 or NRS scores and was compared between groups using the chi‐square test. Continuous variables with normal distribution were compared using the independent samples *t*‐test. Binary logistic regression analysis was performed to assess potential associations between demographic or clinical variables and meaningful pain relief over time. A *p* value < 0.05 was considered statistically significant. To assess whether the early between‐group difference was attributable to baseline imbalance, two sensitivity analyses were performed: a generalized estimating equations (GEE) analysis incorporating baseline score, treatment group, time, group‐by‐time interaction, age, sex, and pain duration with a Gaussian working distribution and robust variance estimation; and a combined logistic regression analysis with treatment allocation included as a covariate alongside baseline pain scores, age, sex, and pain duration.

## 3. Results

The demographic and clinical features of the patients are shown in Table 1. There were no differences between groups regarding age, sex, BMI, and duration of pain (*p* > 0.05). The frequency of systemic disease and hypertension was significantly higher in the ketamine group (*p* = 0.028and *p* = 0.020). The frequency of radiculopathy and TN were similar between groups (*p* > 0.05); therefore, CRPS type 2 was significantly higher in the ketamine group (*p* = 0.002). Pregabalin, gabapentin, duloxetine, and opioid use frequency were similar between groups (*p* > 0.05). Patients who used TCAs were only in the ketamine group (*p* < 0.01). The frequency of using one or two pain medications was similar between groups (*p* > 0.05), but the number of patients using three different medications was higher in the ketamine group (*p* = 0.022). Regarding adverse effects, the proportion of patients experiencing no side effects was significantly lower in the ketamine group compared to the lidocaine group (36.4% vs. 58.6%, *p* = 0.030). Somnolence and dizziness were the most common adverse effects and were similar between groups (*p* > 0.05). Hallucinations were observed exclusively in the ketamine group (22.7%, *p* < 0.01). Psychotomimetic effects including body dysmorphic disorder (9.0%), euphoria (6.8%), and drowsiness (6.8%) were also noted only in the ketamine group. No serious adverse events requiring hospitalization or permanent discontinuation of treatment were recorded in either group (Table 2).

**TABLE 1:** Demographic and clinical features of the patients regarding groups.

		Group 1 (ketamine) (*n* = 44)	Group 2 (lidocaine) (*n* = 58)	*p*
Age (years) (mean ± SD)		51.02 ± 12.53	50.05 ± 16.03	0.744^α^

Sex (*n* %)	Female	27 (61.4)	37 (63.8)	0.838^β^
	Male	17 (38.6)	21 (36.2)	

BMI (kg/m^2^); median (IQR, 25^th^–75^th^)		27.61 (24.27–30.04)	25.35 (22.84–28.26)	0.067^γ^

Systemic diseases^∗^	None	21 (47.7)	39 (67.2)	**0.028** **^β^**
	Hypertension	16 (36.3)	9 (15.5)	**0.020** **^β^**
	Diabetes mellitus	6 (15.6)	7 (12.0)	0.814^β^
	Hypothyroidism	7 (15.9)	9 (15.5)	0.785^β^
	Others	3 (6.8)	6 (10.3))	

Duration of pain (weeks); median (IQR, 25^th^–75^th^)		142.50 (19.00–283.75)	115.00 (41.50–157.00)	0.518^γ^

*Note:* Asterisk represents the total number of patients. IQR: interquartile range. Bold values indicate statistical significance (*p* < 0.05).

Abbreviations: BMI, body mass index; SD, standard deviation.

^α^Independent samples *t*‐test.

^β^Chi‐square test.

^γ^Mann–Whitney *U* test.

**TABLE 2:** Diagnosis, medications, and side effects of the treatment regarding groups.

		Group 1 (ketamine) (*n* = 44)	Group 2 (lidocaine) (*n* = 58)	*p*
Diagnosis	Radiculopathy	19 (43.2)	25 (43.1)	0.994^β^
	CRPS type 2	12 (27.3)	3 (5.2)	**0.002** **^β^**
	TN	7 (15.9)	9 (15.5)	0.957^β^
	Diabetic PNP	3 (6.8)	—	
	Traumatic peripheric nerve lesion	3 (6.8)	3 (5.2)	
	Phantom pain syndrome	—	3 (5.2)	
	Postherpetic neuralgia	—	6 (10.3)	
	Undifferentiated PNP	—	9 (15.5)	

Medications^∗^	Pregabalin	30 (68.1)	30 (51.7)	0.094^β^
	Gabapentin	12 (27.3)	15 (25.8)	0.873^β^
	Duloxetine	24 (54.5)	43 (74.1)	0.136^β^
	TCA	9 (20.4)	—	**<** **0.01** **^β^**
	NSAID	6 (15.6)	21 (36.2)	0.102^β^
	Opioid	6 (15.6)	16 (27.5)	0.147^β^

Number of medications	1	5 (11.3)	13 (22.4)	0.193^β^
	2	18 (40.9)	27 (46.5)	0.688^β^
	3	21 (47.7)	15 (25.9)	**0.022** ^β^
	4	—	3 (5.1)	

Side effects of treatment^∗^	None	16 (36.4)	34 (58.6)	**0.030** **^β^**
	Somnolence	15 (34.0)	12 (25.8)	0.174
	Hallucination	10 (22.7)	—	**<** **0.01** ^β^
	Dizziness	4 (9.03)	7 (12.0)	0.753
	Body dysmorphic disorder	4 (9.0)	—	
	Euphoria	3 (6.8)	—	
	Dryness of the mouth	2 (4.5)	3 (5.2)	
	Drowsiness	3 (6.8)	—	
	Chest tightness	3 (6.8)	2 (3.4)	
	Fatigue	—	1 (1.7)	
	Hypertension	1 (2.3)	—	

*Note:* Asterisk represents the total number of patients; PNP, polyneuropathy; TCA: tricyclic antidepressant; NSAID: nonsteroidal anti‐inflammatory drug. Bold values indicate statistical significance (*p* < 0.05).

Abbreviations: CRPS, complex regional pain syndrome; TN, trigeminal neuralgia.

^β^Chi‐square test.

DN4 scores were similar in groups before treatment; therefore, at the second week, DN4 scores were significantly lower in Group 1 (*p* < 0.01). At the second and sixth months, no significant differences were found between groups (*p* > 0.05). NRS scores were significantly higher in Group 1 before treatment (*p* < 0.01); however, at the second week, NRS scores were significantly lower in Group 1 (*p* < 0.027) (Table 3). DN4 and NRS scores were significantly different for measurements before treatment, at the second week, second month, and sixth month within groups (*p* < 0.01). Two‐way ANOVA could not be performed to detect the effect of the group on scores at different times because of the nonparametric distribution.

**TABLE 3:** Douleur Neuropathique 4 questions scores and Numeric Rating Scale scores between groups at different times of follow‐up.

	Group 1 (ketamine) (*n* = 44)	Group 2 (lidocaine) (*n* = 58)	*p* ^∗^
*Median (IQR, 25–75th)*			
DN4‐before treatment	8.0 (7.0–8.75)	8.0 (7.0–8.0)	0.317
DN4 at 2^nd^ week	4.0 (3.0–5.5)	4.0 (3.0–6.0)	**<** **0.01**
DN4 at the 2^nd^ month	3.0 (2.0–4.0)	4.0 (2.0–7.0)	0.749
DN4 at the 6^th^ month	2.5 (1.0–7.0)	3.0 (1.0–7.0)	0.829
NRS‐before treatment	9.0 (8.0–9.0)	8.0 (7.0–9.0)	**<** **0.01**
NRS at 2^nd^ week	4.0 (2.25–4.0)	4.0 (3.0–6.25)	**0.027**
NRS at the 2^nd^ month	4.0 (2.0–7.0)	4.0 (3.0–8.0)	0.334
NRS at the 6^th^ month	4.0 (1.0–8.0)	2.0 (1.0–8.0)	0.707

*Note:* DN4: Douleur Neuropathique 4 questions; IQR: interquartile range. Bold values indicate statistical significance (*p* < 0.05).

Abbreviation: NRS, Numeric Rating Scale.

^∗^Mann–Whitney U test.

The frequency of meaningful pain relief according to DN4 in Group 1 DN4 was 79.5%, 56.8%, and 59.1% at the second week, second month, and sixth month, respectively. In Group 2, meaningful pain relief frequency was 41.4%, 51.7%, and 53.4% at the second week, second month, and sixth month, respectively. In Group 1, meaningful pain relief was significantly higher at the second week (*p* < 0.01); therefore, there were no differences between groups at the second and sixth months. The frequency of meaningful pain relief, according to NRS, was 86.4%, 61.4%, and 59.1% at the second week, second month, and sixth month, respectively. In Group 2, meaningful pain relief frequency was 50.0%, 53.4%, and 58.6%, respectively. In Group 1, meaningful pain relief was significantly higher at the second week (*p* < 0.01); therefore, there were no differences between groups at the second and sixth months (*p* > 0.05).

Male sex was positively associated with meaningful pain relief in DN4 scores at the sixth month for Group 1 (*p* = 0.006, OR: 16.85). Male sex was also positively associated with meaningful pain relief in NRS scores at the second and sixth months for Group 1 (*p* = 0.041, OR: 6.55, and *p* = 0.006, OR: 26.85, respectively). No association was found between both age and duration of pain with meaningful pain relief in DN4 and NRS scores (*p* > 0.05). Age, sex, and duration of pain were not associated with meaningful pain relief in DN4 and NRS scores at the second week, second month, and sixth month in Group 2 (*p* > 0.05).

Logistic regression analyses were performed separately for each group to identify predictors of meaningful pain relief. The variables included in each model, along with the corresponding odds ratios, 95% confidence intervals, and *p* values, are presented in Tables 4 and 5.

**TABLE 4:** Logistic regression analysis of predictors of meaningful pain relief in Group 1 (ketamine).

Variable	OR	95% CI	*p* value
2nd week—DN4 response			
Age	0.98	0.91–1.05	0.548
Sex	1.13	0.27–4.77	0.866
Pain duration	1.00	0.99–1.00	0.368
2nd week—NRS response			
Age	1.10	0.98–1.23	0.112
Sex	2.99	0.25–35.94	0.387
Pain duration	0.99	0.98–1.00	0.079
2nd month—DN4 response			
Age	1.09	1.01–1.18	**0.032** ^∗^
Sex	2.71	0.58–12.68	0.206
Pain duration	0.99	0.99–1.00	0.072
2nd month—NRS response			
Age	1.03	0.95–1.11	0.467
Sex	6.56	1.08–39.83	**0.041** ^∗^
Pain duration	0.99	0.99–1.00	0.088
6th month—DN4 response			
Age	1.09	1.00–1.20	0.051
Sex	16.86	2.29–124.17	**0.006** ^∗∗^
Pain duration	0.99	0.99–1.00	0.112
6th month—NRS response			
Age	1.09	1.00–1.20	0.051
Sex	16.86	2.29–124.17	**0.006** ^∗∗^
Pain duration	0.99	0.99–1.00	0.112

*Note:* Binary logistic regression. DN4 response: DN4 score < 4; NRS response: ≥ 50% reduction from baseline. Bold values indicate statistical significance.

Abbreviations: CI, confidence interval; OR, odds ratio.

^∗^ *p* < 0.05.

^∗∗^ *p* < 0.01.

**TABLE 5:** Logistic regression analysis of predictors of meaningful pain relief in Group 2 (lidocaine).

Variable	OR	95% CI	*p* value
2nd week—DN4 response			
Age	1.01	0.97–1.05	0.502
Sex	4.49	1.31–15.37	**0.017** ^∗^
Pain duration	0.99	0.99–1.00	0.282
2nd week—NRS response			
Age	0.99	0.96–1.03	0.701
Sex	1.57	0.49–5.01	0.449
Pain duration	0.99	0.99–1.00	0.125
2nd month—DN4 response			
Age	1.02	0.98–1.06	0.342
Sex	2.21	0.69–7.04	0.181
Pain duration	0.99	0.98–1.00	0.155
2nd month—NRS response			
Age	1.01	0.97–1.05	0.715
Sex	2.31	0.69–7.70	0.172
Pain duration	0.99	0.98–1.00	0.108
6th month—DN4 response			
Age	0.99	0.96–1.03	0.782
Sex	1.00	0.28–3.50	0.995
Pain duration	0.99	0.98–1.00	**0.011** ^∗^
6th month—NRS response			
Age	1.01	0.97–1.05	0.510
Sex	1.58	0.47–5.32	0.456
Pain duration	0.99	0.98–1.00	0.096

*Note:* Binary logistic regression. DN4 response: DN4 score < 4; NRS response: ≥ 50% reduction from baseline. Bold values indicate statistical significance.

Abbreviations: CI, confidence interval; OR, odds ratio.

^∗^ *p* < 0.05.

In the GEE sensitivity analysis, the ketamine group continued to show significantly lower NRS scores after adjustment for baseline covariates (*β* = −2.39, 95% CI: −3.55 to −1.23, *p* < 0.001). The group‐by‐time interaction did not reach statistical significance (*β* = 0.63, *p* = 0.051), though a borderline trend toward convergence of scores between groups over time was observed. For DN4 scores, the between‐group difference similarly persisted after adjustment (*β* = −1.97, 95% CI: −2.97 to −0.98, *p* < 0.001), with a significant group‐by‐time interaction (*β* = 0.60, *p* = 0.035), indicating gradual attenuation of ketamine’s early advantage over time.

To determine whether ketamine’s early advantage reflected a genuine treatment effect rather than baseline differences between groups, a combined logistic regression analysis was conducted with treatment group included as a predictor. After adjustment for baseline NRS score, age, sex, and pain duration, ketamine group membership remained a significant independent predictor of meaningful pain relief at 2 weeks for both NRS (OR = 7.23, 95% CI: 2.11–24.72, *p* = 0.002) and DN4 (OR = 3.25, 95% CI: 1.31–8.09, *p* = 0.011) outcomes. At 2 and 6 months, the treatment group was no longer a significant predictor for either measure (all *p* > 0.05), in keeping with the comparable efficacy observed between groups at midterm follow‐up. Full results are presented in Table 6.

**TABLE 6:** Combined logistic regression analysis of predictors of meaningful pain relief (both groups).

Variable	OR	95% CI	*p* value
2nd week—NRS response			
Treatment (ketamine vs lidocaine)	7.23	2.11–24.72	**0.002** ^∗∗^
Baseline NRS	1.80	1.06–3.04	**0.029** ^∗^
Age	1.02	0.98–1.06	0.292
Sex (male vs female)	2.52	0.80–7.99	0.116
Pain duration	0.99	0.99–1.00	**0.036** ^∗^
2nd week—DN4 response			
Treatment (ketamine vs lidocaine)	3.25	1.31–8.09	**0.011** ^∗^
Baseline DN4	0.77	0.55–1.09	0.147
Age	1.01	0.97–1.04	0.709
Sex (male vs female)	2.39	0.94–6.07	0.066
Pain duration	1.00	0.99–1.00	0.102
2nd month—NRS response			
Treatment (ketamine vs lidocaine)	2.41	0.89–6.51	0.082
Baseline NRS	0.61	0.37–0.99	**0.045** ^∗^
Age	1.00	0.96–1.03	0.838
Sex (male vs female)	2.56	0.93–7.06	0.068
Pain duration	0.99	0.99–1.00	**0.014** ^∗^
2nd month—DN4 response			
Treatment (ketamine vs lidocaine)	1.30	0.55–3.06	0.552
Baseline DN4	0.87	0.63–1.22	0.422
Age	1.03	1.00–1.07	0.065
Sex (male vs female)	1.95	0.78–4.87	0.151
Pain duration	1.00	0.99–1.00	0.066
6th month—NRS response			
Treatment (ketamine vs lidocaine)	1.47	0.57–3.80	0.423
Baseline NRS	0.62	0.39–1.00	0.050
Age	1.01	0.98–1.05	0.431
Sex (male vs female)	2.65	0.95–7.38	0.063
Pain duration	1.00	0.99–1.00	**0.022** ^∗^
6th month—DN4 response			
Treatment (ketamine vs lidocaine)	1.53	0.62–3.75	0.356
Baseline DN4	1.01	0.72–1.42	0.947
Age	1.01	0.97–1.04	0.760
Sex (male vs female)	2.23	0.86–5.77	0.098
Pain duration	0.99	0.99–1.00	**0.020** ^∗^

*Note:* Binary logistic regression. Both treatment groups combined. NRS response: ≥ 50% reduction from baseline NRS score; DN4 response: DN4 score < 4. Pain duration expressed in weeks. Sex coded as 1 = female, 2 = male. Bold values indicate statistical significance.

Abbreviations: CI, confidence interval; OR, odds ratio.

^∗^ *p* < 0.05.

^∗∗^ *p* < 0.01.

## 4. Discussion

In this retrospective study, the analgesic efficacy and safety profiles of intravenous ketamine and lidocaine infusion therapies were compared in patients with chronic peripheral NP. The principal finding was that ketamine infusion was associated with a greater early analgesic response at 2 weeks, whereas both treatment modalities demonstrated comparable efficacy at midterm (2 and 6 months) follow‐up.

Ketamine has been widely used in chronic pain management due to its ability to reduce central sensitization and opioid tolerance [12, 13]. However, there is no consensus regarding the optimal dose, duration, or infusion protocol. In a Delphi survey, Voute et al. reported that intravenous ketamine doses ranging from 0.5 to 0.9 mg/kg/day administered for 4 days were preferred [14]. In patients with complex regional pain syndrome, Schwartzman et al. demonstrated that ketamine infused at 0.35 mg/kg/h for 4 h daily over 10 days resulted in significant improvements in pain and quality of life at 3 months compared with placebo [15]. The analgesic benefit observed in our study at subanesthetic doses is consistent with these findings. Moreover, the shorter infusion duration and treatment period used in our protocol suggest that meaningful analgesia may be achieved with lower cumulative doses of ketamine (< 1.2 mg/kg), which may offer practical advantages in routine clinical settings. The dosing regimens used in this study warrant some discussion. The ketamine dose of 1 mg/kg/hour per session falls at the midpoint of the 0.5–2 mg/kg/h subanesthetic range recommended by the ASRA/AAPM/ASA consensus guidelines [7], rather than at the higher end as previously stated. We acknowledge that this regimen does not meet the guideline’s recommendation for sessions lasting more than 2 h; however, the 5‐day consecutive protocol resulted in a total cumulative dose of 5 mg/kg, which was considered appropriate for an outpatient setting. For lidocaine, Kim et al. used the same dose of 3 mg/kg over 1 h and demonstrated short‐term efficacy with repeated infusions, though without sustained pain relief at follow‐up [16]. While higher doses near 5 mg/kg may yield more consistent results in some studies [10], the 3 mg/kg dose was selected to maintain a safe infusion rate in our clinic. We recognize that this dosing asymmetry between groups is a limitation of the study. Whether the earlier plateau in lidocaine’s analgesic effect reflects a true difference in dose–response trajectories between the two agents remains an open question that prospective studies with pharmacologically matched protocols would be better placed to address.

Previous reviews have shown that ketamine provides short‐term analgesic benefits in noncancer chronic pain, although its long‐term efficacy remains controversial. Michelet et al. reported that ketamine was effective for up to 12 weeks, but psychomimetic adverse effects increased with higher doses and longer treatment durations [17]. Consistent with these findings, hallucinations were observed more frequently in the ketamine group in our study; however, these adverse effects were mild to moderate and did not necessitate treatment discontinuation. Nevertheless, the psychogenic side effects of ketamine should be carefully considered, particularly in patients with psychiatric comorbidities or those receiving neuropsychiatric medications. In line with previous reports, studies involving patients with failed back surgery syndrome and post‐thoracotomy pain have demonstrated limited long‐term efficacy of ketamine infusion [18, 19]. Similarly, a meta‐analysis including 211 patients with refractory chronic NP reported that intravenous ketamine provided only short‐term analgesic benefit [20].

Intravenous lidocaine infusion has been reported as a relatively safe treatment option with a favorable side‐effect profile in various NP conditions, including complex regional pain syndrome and postherpetic neuralgia [16, 21, 22]. However, the duration of analgesic effect has generally been described as short. Moulin et al. found no significant difference between placebo and intravenous lidocaine (5 mg/kg) in terms of pain relief and quality of life over a 4‐week follow‐up in patients with chronic peripheral NP [23]. In contrast, our study demonstrated clinically meaningful pain relief, defined as a ≥ 50% reduction in DN4 and NRS scores, persisting up to 6 months following lidocaine infusion. These findings may reflect differences in patient populations, infusion protocols, or concomitant treatments. In a retrospective study of 81 patients receiving repeated lidocaine infusions, transient dizziness was the most commonly reported adverse effect, and no association was found between pain duration and treatment efficacy [24]. Similarly, we observed no correlation between age or pain duration and treatment response in either group.

In the present study, lidocaine infusion was associated with fewer adverse effects compared with ketamine infusion. The most frequently observed side effects were dizziness, somnolence, and dry mouth, which were generally mild and self‐limiting. Liu et al. reported that intravenous lidocaine improved pain, anxiety, depression, and quality of life for up to 4 weeks following a single infusion in patients with postherpetic neuralgia [25]. The more pronounced early analgesic response observed in the ketamine group may be partially explained by the interaction between pain intensity, mood, and affective symptoms. Ketamine has been shown to modulate glutamatergic neurotransmission and exert rapid antidepressant and anxiolytic effects, which may contribute to early pain relief [26].

Direct comparisons between ketamine and lidocaine infusion in chronic NP are limited, as most comparative studies have focused on acute or postoperative pain. In a study comparing ketamine and lidocaine infusions following open nephrectomy, lidocaine was associated with lower postoperative pain scores and a reduced risk of developing NP at 3 months, whereas ketamine was not effective and was associated with more adverse effects [27]. In another small study involving patients with spinal cord injury and refractory NP, ketamine reduced pain intensity, whereas lidocaine and placebo were ineffective [28]. Our findings add to this limited body of evidence by providing comparative real‐world data in a heterogeneous chronic peripheral NP population.

The strengths of this study include the comparison of two commonly used infusion therapies using validated pain assessment tools and a follow‐up period extending to 6 months. However, several limitations should be acknowledged. The retrospective design and the inclusion of patients with heterogeneous NP etiologies may have influenced the generalizability of the findings. Serum ketamine and lidocaine levels were not measured, which limits pharmacokinetic interpretation of the treatment responses observed. Baseline differences between groups, including a higher frequency of CRPS type 2, systemic comorbidities, and TCA use in the ketamine group, should be kept in mind when interpreting the between‐group comparisons, although the sensitivity analyses confirmed that the early treatment effect remained robust after adjustment for these variables. Finally, the dosing asymmetry between arms means that the two regimens were not pharmacologically matched; conclusions regarding comparative dose‐response relationships or ceiling effects should therefore be treated with caution.

## 5. Conclusion

Overall, ketamine infusion demonstrated a greater early analgesic response compared with lidocaine infusion in patients with chronic peripheral NP, while both treatments showed comparable efficacy at midterm follow‐up (2 and 6 months). Although ketamine may offer short‐term advantages in selected patients, lidocaine appears to be associated with a more favorable safety profile. These findings may help inform clinical decision‐making and support future prospective studies evaluating infusion therapies for NP.

## Author Contributions

Conceptualization: Mevlüt Gökhan Sucu; methodology: Ayşegül Akyüz Yildirim; formal analysis and investigation: Ayşegül Akyüz Yildirim; data curation: Tuğçe Yavuz Mollavelioğlu; writing–original draft preparation: Tuğçe Yavuz Mollavelioğlu; writing–review and editing: Mevlüt Gökhan Sucu, Nalan Çelebi; supervision: Nalan Çelebi.

## Funding

This research received no specific grant from funding agencies in the public, commercial, or not‐for‐profit sectors.

## Disclosure

Part of this study was previously presented as an oral presentation at the 18th National Pain Congress (Turkiye). All authors read and approved the final manuscript and agree to be accountable for all aspects of the work.

## Ethics Statement

This study was conducted in accordance with the principles of the Declaration of Helsinki (1964) and its later amendments. Ethical approval was obtained from the Ethics Committee of Hacettepe University Medical School (approval date: May 10, 2022; No. 2022/08‐45). Written informed consent was obtained from all participants prior to their inclusion in the study.

## Conflicts of Interest

The authors declare no conflicts of interest.

## Data Availability

The data supporting the findings of this study are available from the corresponding author upon reasonable request.
